# Supplementary material for: Comparative proteomics analysis reveals the molecular mechanism of enhanced cold tolerance through ROS scavenging in winter rapeseed (Brassica napus L.)
Source: PLoS One. 2021 Jan 12;16(1):e0243292. doi: 10.1371/journal.pone.0243292 (PMC7802968; doi:10.1371/journal.pone.0243292)
Supplement: Attachment — Submitted filename: Response to Reviewers.docx [file pone.0243292.s007.docx]

**Author's response to review comments**

First of all, and thanks the scholars who reviewed this manuscript for their valuable comments on this paper, thanks the responsible editor of the manuscript for your letter, as well as the important guiding significance to our researches. We have carefully revised and checked the manuscript in accordance with the review comments to make the paper more complete and meet the requirements of the journal.

**Reviewer #1:**

In this manuscript, the authors reported two winter rapeseed cultivars, cold tolerant and cold sensitive, were used to reveal the morphological, physiological, and proteomic characteristics in leaves of plants after treatment at different temperature points. The candidate pathways of DAPs involved were analyzed and ROS scavenging pathway may play function in rapeseed cold tolerance. The paper is poorly displayed and following items should be modified.

1. The format of references is incorrect, please correct it.

RESPONSE: We have revised the format of the references in accordance with the requirements of the journal.

2. The description of Result 2.2, “…identified based on the criteria of p < 0.005 for the adjusted p-value…”, is confused.

RESPONSE: The sentence “…identified based on the criteria of p < 0.005 for the adjusted p-value…” have been revised to “…identified based on the criteria of p < 0.05 for the adjusted p-value…”.

3. An obvious descriptive error existed in Methods 4.1, “…store at -80 reaching concentrations thaEach sample….”.

RESPONSE: The sentence “then immediately frozen in liquid nitrogen and stored at -80 reaching concentrations thaEach sample was pooled from three plants and three biological replicates were included.” have been revised to “then immediately frozen in liquid nitrogen and stored at -80 that each sample was pooled from three plants and three biological replicates were included.”

4. Abstract part, the first line, it is should be “cultivars” not “culvers”. It is recommended to find native English speaker to modify the language of the paper.

RESPONSE: The word “culvers” have been revised to “cultivars”.

5. It is confused to understand if the proteomics performed with biological repetition?

RESPONSE: We used 3 sets of biological replicates when using RNA sequencing.

**Reviewer #2:**

In this work, the authors performed comparative proteomics analysis reveals the molecular mechanism of enhanced cold tolerance through ROS scavenging in winter rapeseed (Brassica napus L.). Although this work is interesting, the manuscript is not well organized, and the data are not well presented. Therefore, the paper needs to be thoroughly revised.

1. The authors must carefully proof-read the manuscript to minimize typographical, grammatical, and bibliographic errors. Some typographical errors, some nonsensical or confusing sentences should be corrected.

RESPONSE: We have found a professional company to polish the article.

2. In Abstract, “Two winter rapeseed culvers, “NF” (cold tolerant) and “NS” (cold sensitive), were used to reveal the morphological, physiological, and proteomic characteristics……”. What does ‘culvers’ mean? ‘culvers’ or ‘cultivars’? Please check the spelling.

RESPONSE: The word “culvers” have been revised to “cultivars”.

3. In Abstract, authors refer to two winter rapeseed culvers “NF” and “NS”, among, “NF” represent cold-tolerant cultivar and “NS” represent cold-sensitive cultivar. However, in Introduction and Methods parts, authors refer to “strong cold-tolerant variety “NS” and '17NTS57' (abbreviated as “NS”, cold-tolerant…) and 'NQF24' (abbreviated as “NF”, cold-sensitive…) winter rapeseed (Brassica napus L.). These seem to be particularly controversial. Please confirm and explain why?

RESPONSE: It's my fault. We have interchanged "NF" and "NS" in the abstract.

4. In Plant materials, cold stress treatment and physiological part, authors say “the cold-tolerant variety ‘17NTS57’ beyond 90% survival rate under −26 °C of extreme low temperature during overwintering, please provide data supporting the result or cite the related references.

RESPONSE: We identified the cold resistance of these two varieties in Zhangye city, Gansu Province in 2018. We will attach the relevant data below.

5. In results part, ‘(figure2B )’ should put it after the sentence ‘By comparing all of the DAPs between the two varieties at three treated time points, the Venn diagram shows the number of more- or less-abundant DAPs as well as specific and commonly abundant DAPs in the different groups.’

RESPONSE: We have revised the manuscript in accordance with the opinions of the reviewers.

6. In general, the technical approaches of RT-qPCR seem fine. However, statistics for figure 6 are not given. Also, statistics for DAGs (real-time quantitative RT-PCR) abundance changes (eg p-values) are not given (not in method and not in legend). What method were used? ANOVA? One or two way? What was the level of significance?

RESPONSE: According to revise comments, we redid figure 6, as follows.

Figure 6. Transcriptional level of selecting corresponding genes of DAPs between two varieties under cold stress. The datas were shown as averages of three independent biological replicates±SD.*P<0.05,**P<0.01,***P<0.001.

7. Authors selected 23 DAPs for detecting the mRNA levels using qRT-PCR. However, the protein names are not given in figure 6 or in Result part. The protein names are replaced by Numbers 1, 2, 3, 4, 5……

RESPONSE: According to revise comments, we have made a table S1 of candidate protein names, as follows.

Table S6 The candidate proteins information

| Number | Protein ID | Protein name |
| --- | --- | --- |
| 1 | XP_013728819.1 | trpD; anthranilate phosphoribosyltransferase |
| 2 | XP_013676058.1 | trpD; anthranilate phosphoribosyltransferase |
| 3 | XP_013722305.1 | trpD; anthranilate phosphoribosyltransferase |
| 4 | XP_013718039.2 | TAT; tyrosine aminotransferase |
| 5 | XP_013657201.1 | trpG; anthranilate synthase component II |
| 6 | XP_022545953.1 | trpG; anthranilate synthase component II |
| 7 | XP_022565834.1 | aroB; 3-dehydroquinate synthase |
| 8 | XP_013661385.1 | aroB; 3-dehydroquinate synthase |
| 9 | XP_013640777.1 | aroDE, DHQ-SDH; 3-dehydroquinate dehydratase / shikimate dehydrogenase |
| 10 | XP_013726187.1 | GOT1; aspartate aminotransferase, cytoplasmic |
| 11 | XP_013721995.1 | GOT1; aspartate aminotransferase, cytoplasmic |
| 12 | XP_013664180.1 | PAT, AAT; bifunctional aspartate aminotransferase and glutamate/aspartate-prephenate aminotransferase |
| 13 | XP_013707917.2 | PAT, AAT; bifunctional aspartate aminotransferase and glutamate/aspartate-prephenate aminotransferase |
| 14 | XP_022571603.1 | CYP73A; trans-cinnamate 4-monooxygenase |
| 15 | XP_022557738.1 | CYP73A; trans-cinnamate 4-monooxygenase |
| 16 | XP_013683255.1 | CYP73A; trans-cinnamate 4-monooxygenase |
| 17 | XP_013678442.1 | FLS; flavonol synthase |
| 18 | XP_022562120.1 | FLS; flavonol synthase |
| 19 | XP_013715671.1 | FLS; flavonol synthase |
| 20 | XP_013672989.1 | ALDH7A1; aldehyde dehydrogenase family 7 member A1 |
| 21 | XP_013707249.1 | ALDH; aldehyde dehydrogenase (NAD+) |
| 22 | XP_013745666.1 | ALDH; aldehyde dehydrogenase (NAD+) |
| 23 | XP_013723238.1 | HPD, hppD; 4-hydroxyphenylpyruvate dioxygenase |

8. In Result part, no any information and sentences related to figure 7. Please add …..!

RESPONSE: According to revise comments, we have added the information of figure 7 in the results.

9. The results lack systematic in-depth analyses. Authors think cold-tolerant rapeseed enhance cold tolerance through ROS scavenging, but no related candidate proteins were in-depth analyzed and studied.

RESPONSE: According to revise comments, we have added relevant information to the results.

10. Figure quality is too low and the legends need to be reorganized and rephrased.

RESPONSE: According to revise comments, we have made the corresponding changes
