# Supplementary material for: Comparative proteomics analysis reveals the molecular mechanism of enhanced cold tolerance through ROS scavenging in winter rapeseed (Brassica napus L.)
Source: PLoS One. 2021 Jan 12;16(1):e0243292. doi: 10.1371/journal.pone.0243292 (PMC7802968; doi:10.1371/journal.pone.0243292)
Supplement: Attachment — Submitted filename: Response to Reviewers.docx [file pone.0243292.s008.docx]

**Author's response to review comments**

First of all, and thanks the scholars who reviewed this manuscript for their valuable comments on this paper, thanks the responsible editor of the manuscript for your letter, as well as the important guiding significance to our researches. We have carefully revised and checked the manuscript in accordance with the review comments to make the paper more complete and meet the requirements of the journal.

**Reviewer #1:**

The authors modified all the concerns we raised, I think it can be accepted with a minor revision.

1. Please write the full name for the abbreviation of AVG when it first appeared.

RESPONSE: According to revise comments, we have made the corresponding changes

2. In methods part, the real-time quantitative PCR, “The Actin gene was used as an internal reference…..”, the accession number for the actin gene used should be listed in the paper.

RESPONSE: According to revise comments, we have increased the accession number of the actin gene
